# Dietary purslane (*Portulaca oleracea L.*) promotes the growth performance of broilers by modulation of gut microbiota

**DOI:** 10.1186/s13568-021-01190-z

**Published:** 2021-02-23

**Authors:** Cong Wang, Qing Liu, Fengchun Ye, Hongbo Tang, Yanpeng Xiong, Yongfei Wu, Luping Wang, Xuanbiao Feng, Shuiyin Zhang, Yongmei Wan, Jianhua Huang

**Affiliations:** 1 School of life science, Jiangxi Science and Technology Normal University, 330013 Nanchang, China; 2 Jiangxi Key Laboratory of Bioprocess Engineering, Jiangxi Science and Technology Normal University, 330013 Nanchang, China; 3 Jiangxi Yifeng County Qiaoxi Veterinary Station, 336300 Yifeng, Jiangxi China

**Keywords:** Purslane, Sanhuang broilers, Growth performance, Gut microbiota, Carbohydrate metabolism

## Abstract

Purslane is a widespread wild vegetable with both medicinal and edible properties. It is highly appreciated for its high nutritional value and is also considered as a high-quality feed resource for livestock and poultry. In this study, Sanhuang broilers were used to investigate the effect of feeding purslane diets on the growth performance in broilers and their gut microbiota. A total of 48 birds with good growth and uniform weight were selected and randomly allocated to four treatment groups A (control), B, C and D. Dietary treatments were fed with basal diet without purslane and diets containing 1%, 2% and 3% purslane. The 16S rDNA was amplified by PCR and sequenced using the Illumina HiSeq platform to analyze the composition and diversity of gut microbiota in the four sets of samples. The results showed that dietary inclusion of 2% and 3% purslane could significantly improve the growth performance and reduce the feed conversion ratio. Microbial diversity analysis indicated that the composition of gut microbiota of Sanhuang broilers mainly included *Gallibacterium*, *Bacteroides* and *Escherichia-Shigella*, etc. As the content of purslane was increased, the abundance of *Lactobacillus* increased significantly, and *Escherichia-Shigella* decreased. LEfSe analysis revealed that *Bacteroides_caecigallinarum*, *Lachnospiraceae*, *Lactobacillales* and *Firmicutes* had significant differences compared with the control group. PICRUSt analysis revealed bacteria mainly enriched in carbohydrate metabolism pathway due to the additon of purslane in the diet. These results suggest that the addition of purslane to feed could increase the abundance of *Lactobacillus* in intestine, modulate the environment of gut microbiota and promote the metabolism of carbohydrates to improve its growth performance. This study indicates that the effect of purslane on the growth-promoting performance of broilers might depend on its modulation on gut microbiota, so as to provide a certain scientific basis for the application of purslane in the feed industry.

**Supplementary Information:**

The online version contains supplementary material available at 10.1186/s13568-021-01190-z.

## Introduction

Sanhuang broiler chicken, also is known as triple-yellow chicken, is one of the most famous breeds of poultry as well as native chickens in China, named for its yellow beak, yellow feather and yellow claws. The meat of Sanhuang chicken is tender, delicious and nutritious, and contains high protein and amino acid content, but low fat content. Sanhuang chicken is also a great source of trace elements such as iron, copper and zinc, and is rich in vitamins, which can be used to nourish blood and keep healthy. In the livestock and poultry breeding industry, the performance of livestock and poultry is an important factor affecting economic benefits. However, slow growth is another characteristic of Chinese local breeds. Some studies have indicated that added purslane to the feed of broiler chickens has a good effect on enhanced the appetite of animals or promoted the growth performance (Habibian et al. 2019; Kartikasari et al. 2018).

Purslane (*Portulaca oleracea L.*) is an annual herb that is widely distributed around the globe. Purslane is a nutritious vegetable with high antioxidant properties. It is rich in nutrients such as glucose and malic acid, and is also recognized as the richest source of α-linolenic acid, essential omega-3 fatty acids, ascorbic acid, α-tocopherol and β-carotene (Uddin et al. 2014). Omega-3 polyunsaturated fatty acids (PUFAs) are essential nutrients for health benefits such as reducing the risk of coronary heart disease and depression, regulating metabolic syndrome, and relieving anxiety (Schunck et al. 2018; Shahidi and Ambigaipalan 2018). Purslane contains bioactive compounds like flavonoids, alkaloids, polysaccharides, etc., which have a variety of pharmacological effects, such as antibacterial, antioxidant, hypoglycemic, hypolipidemic, neuroprotection and so on (Rahimi et al. 2019; Zhou et al. 2015). The edible and medicinal value of purslane is of momentous significance to the growth and development of organisms, and it has shown great nutritional potential and has very far-reaching application prospects in the future.

The gut microbiota is essential for the health of the host, for example, to promote host metabolism, defense against pathogens, enhance immunity, etc., and directly or indirectly affect most of the host’s physiological functions. Meanwhile, the intestinal flora is involved in metabolic diseases such as obesity and dyslipidemia of the organism. The interplay of the gut microbiota with host can be mediated by diet, and its effects starts but is far beyond the intestine (Dabke et al. 2019). At present, antibiotics are widely used in animal husbandry and weight gain by gut microbiota manipulation to improve the growth performance of animals (Angelakis 2017). Although they have specific benefits to the health of animals, some pathogens develop resistance to them quickly, and if the abuse of antibiotics in livestock feed may lead to the transfer of drug-resistant pathogens to humans. Therefore, it is necessary to develop new products as a substitute for antibiotics in feed to better promote the health and growth performance of poultry, which is also a major guarantee for the health of humans who consume such animal products.

Traditional Chinese herbal medicine contains numerous biologically active compounds, which can provide various nutritional and health benefits to animals, so it can be used as a feed additive (Abdallah et al. 2019). As a feed additive, purslane is especially worthy of attention in enhancing nutrition, regulating metabolism and promoting the digestion and absorption of nutrients in animals. This study aims to investigate the effect of purslane on the growth performance and gut microbiota in broiler chickens, as well as the role of these intestinal bacteria in regulating the growth and metabolism. In practical application, it is possible to achieve the purpose of partially replacing antibiotics and gaining weight, which provides a certain scientific basis for the application of purslane in animal feed industry.

## Materials and methods

### Feeding management and diets

The experiment was carried out in a chicken farm in Ji’an City, Jiangxi Province, China, and it lasted for 30 days from November 5 to December 4, 2019. A total of 48 Sanhuang chickens with good growth and body weight of 285 ± 30 g were selected and randomly allocated to four treatment groups A (control), B, C and D, with 3 replicates in each group and 4 chickens in each replicate. During the entire test period, the designated personnel took up the responsibility to manage tasks such as feeding, drinking and cleaning. Infrared lamp was used for heating, the temperature was maintained at 19–28 °C, and normal ventilation and lighting were kept. Fed once in the morning and afternoon, and eat freely throughout the test period.

The dietary used were all powdered feeds, and the basic diet consisted mostly of corn and soybean meal. The detailed dietary and nutritional composition is presented in Additional file 1: Table S1. The A group was fed only the basal diet, and the diets of the B, C and D groups were supplemented with 1%, 2% and 3% purslane, respectively. All of purslane were collected from the wild area and ground into powder after washed and dried for feeding trials.

### Sample collection, DNA extraction and amplification

On the last day of the test, the final weight of each chicken was measured and three fecal samples were collected from each group. A total of twelve fecal samples were stored in different sterile cryotube and placed in liquid nitrogen, then brought back and transferred to the − 80 ℃ refrigerator for storage until further analysis.

Genomic DNA of all feces samples were extracted according to the specifications of kit. The concentration and purity of DNA were detected by NanoDrop 2000 (Thermo, USA), while the quality and integrity of DNA were detected by 1 % agarose gel electrophoresis. The V3 + V4 region of bacterial 16S rDNA was amplified by PCR with the specific primers, where the primers were designed according to the conserved region, and then sequencing adapters were added to the end of primers for PCR amplification. PCR amplification was conducted under the following conditions: 5 min at 94 °C for initialization, 30 s denaturation at 94 ℃ for 30 cycles, 30 s annealing at 52 ℃, and 30 s extension at 72 ℃, and a final 10 min elongation at 72 ℃.

### Sequencing and data processing

After PCR amplification, the products were purified, quantified and homogenized to get a sequencing library. Then library quality control was performed for constructing libraries, qualified libraries were paired-end sequenced by Illumina HiSeq 2500 platform of Biomarker Technologies (Beijing, China). According to overlap relation between paired-end reads, FLASH software (v1.2.11) (Magoc and Salzberg 2011) was used to merge the paired reads into a sequence and to filter out nonconforming tags in order to collect the raw tags. The minimum overlap length was 10 bp and the maximum error ratio was 0.2. Trimmomatic software (v0.33) (Bolger et al. 2014) was used to filter merged raw tags to get high quality clean tags. The sliding window was 50 bp, and the average quality in sliding window was 20. Finally, UCHIME software (v8.1) (Edgar et al. 2011) was used to identify and remove chimera sequences to get effective tags.

### Microbial community and diversity analysis

The USEARCH software (v10.0) (Edgar 2013) was used to cluster all effective tags for all samples at 97 % similarity level. In general, if the similarity between sequences is higher than 97 %, it can be defined as an operational taxonomic unit (OTU), and each OTU corresponds to a representative sequence. The OTU was filtered when species abundance less than 0.005 % (Bokulich et al. 2013). Then the Ribosomal Database Project (RDP) classifier (Wang et al. 2007) was used to classify OTUs under the 80 % threshold and perform taxonomic annotation for OTU on the basis of Silva (Bacteria) (Release132, http://www.arb-silva.de) (Quast et al. 2013) taxonomic database.

The QIIME software (v1.9.1) (Caporaso et al. 2010) was used to count the community composition of each sample and generate the species abundance table at different taxonomic levels, and then use R language tool to draw community structure graph of samples at different taxonomic levels. Furthermore, QIIME software was used to select OTU sequences with the highest abundance at genus taxonomic level as the representative sequence to perform multiple sequences alignment and phylogenetic tree construction, and then the graph was drawn through the Python language tool. With the phylogenetic relationship and relative abundance information of each OTU in the samples, the species annotation results of a single sample was visualized by KRONA software (Ondov et al. 2011). According to the taxonomic database of existing microbial species provided by NCBI, MEGAN5 software (Huson et al. 2007) was used to return the species abundance information obtained by sequencing to the taxonomic system relation tree of the database to understand the evolutionary relationships and abundance differences of all microorganisms in the samples. According to the OTU abundance table, Mothur software (v1.30) (Schloss et al. 2009) was used to evaluate the Alpha diversity and the Chao1 and Shannon indices were calculated to compare the differences in species abundance and diversity between groups (Chao et al. 2006).

### LEfSe analysis and PICRUSt prediction

The linear discriminant analysis (LDA) effect size (LEfSe) analyses were performed on the website http://huttenhower.sph.harvard.edu/galaxy (Segata et al. 2011). According to the established Biomarker screening criteria (LDA score > 4), the eligible biomarkers were identified. Functional capacity of gut microbiota was predicted using the Phylogenetic Investigation of Communities by Reconstruction of Unobserved States (PICRUSt) (Parks et al. 2014). First, the generated OTU-table needs to be standardized. Then, according to the unique greengene id corresponding to each OTU, the KEGG family information related to OTU can be obtained, so as to calculate the abundance of KEGG (Kyoto Encyclopedia of Genes and Genomes) and compare it with KEGG database to obtain functional predictions.

### Statistical analysis

The statistical analysis of all recorded data in the experiment was carried out by R statistical software. One-way analysis of variance (ANOVA) was used for multiple samples, and the differences between groups were examined by two-tailed Student’s t-test. The G-test and Fisher test methods in STAMP were used to test the significant differences in species abundance between different samples, and pairwise T-tests was applied between different groups. Test results of P < 0.05 were considered significant, and P < 0.01 were considered extremely significant.

## Results

### Effect of purslane on the growth performance of Sanhuang chickens

In this study, we randomly divided 48 Sanhuang chickens into 4 groups with 12 chickens in each group. To investigate the effect of purslane on growth performance, we recorded the initial and final weight of each chicken and measured the daily intake of each group. The four groups had no significant difference regarding initial weight.

**Fig. 1:**
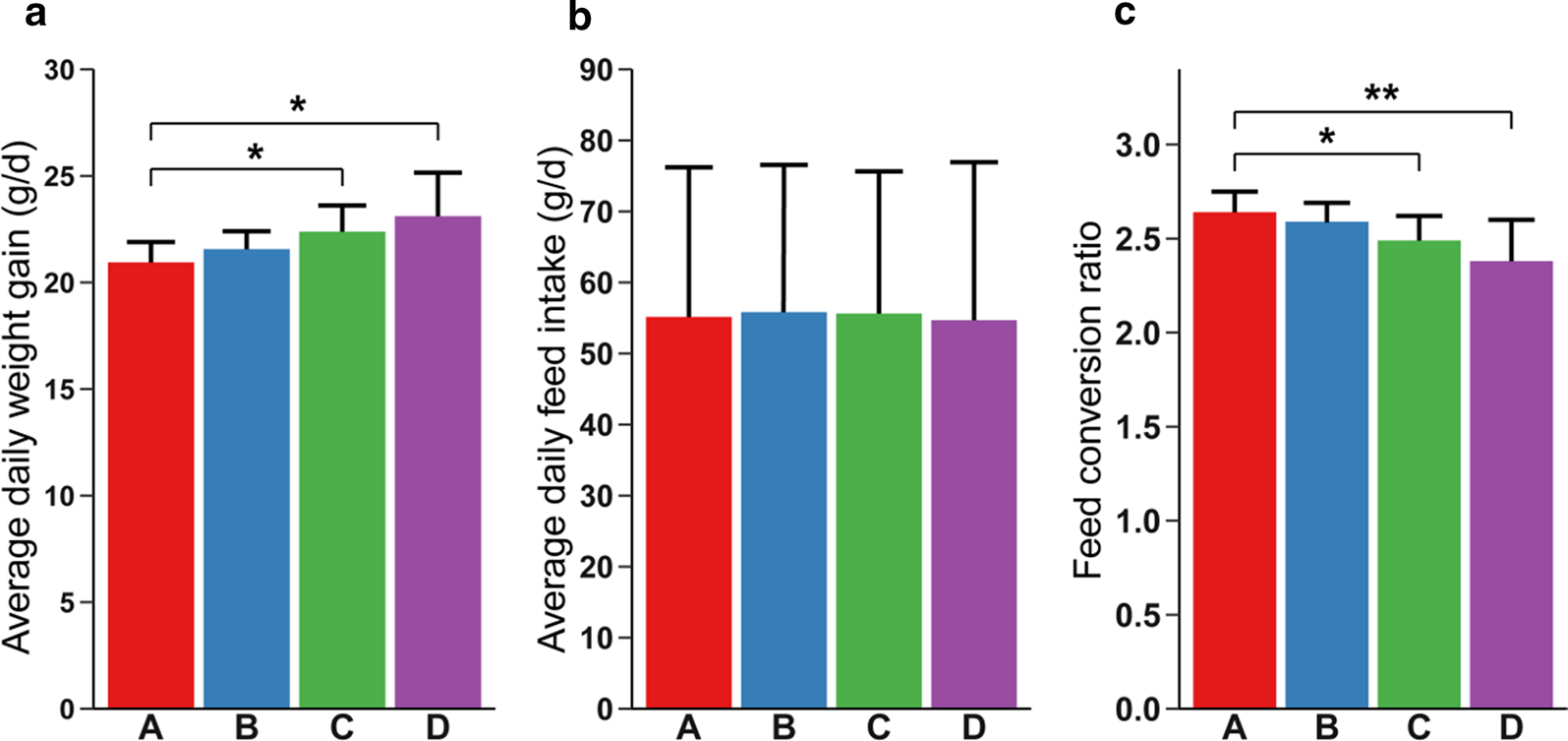
Effects of different levels of purslane on growth performance of broilers. A, B, C and D correspond to the four treatment groups with 0%, 1%, 2% and 3% purslane, respectively. Average daily weight gain (**a**), average daily feed intake (**b**) and feed conversion ratio (**c**) were compared among the four treatment groups. Asterisk indicates significantly changed (p < 0.05), double asterisk indicates extremely significantly changed (p < 0.01)

After the end of the test period, we compared the average daily food intake and weight gain of each group, and the changes in feed conversion ratio (FCR) were analyzed (Fig. 1). There was no significant difference in the daily food intake between the four groups (P > 0.05), but the average daily weight gain of groups C and D with 2 % and 3 % purslane was significantly higher than that of the control group A (P < 0.05), which increased by 1.44 g and 2.17 g, respectively. Subsequently, the analysis results of FCR suggested that it showed a gradual decrease trend with the increase of purslane. Among them, the FCR of the C and D groups decreased significantly, decreased by 0.15% and 0.26%, respectively, especially in group D, the decline was extremely significant (p < 0.01). These results indicate that the addition of purslane to feed broiler chickens has no significant effect on feed intake, but gains weight significantly, achieves the purpose of improving growth performance and reducing FCR. In the meantime, our results demonstrate that the best effect of reducing the FCR was 3 % purslane-fed.

### Assessment of sequencing data quality and OTU analysis of fecal samples

A total of 937,475 raw tags were obtained from 12 fecal samples. After filtering a series of low-quality raw tags and removing the chimera sequences, 837,028 effective tags were obtained for subsequent analysis, and the average number of high-quality sequences for each sample was 69,752. The length distribution range of effective tags was 350–450 bp, and the average length is 423 bp. The average GC content accounts for 52.48 %, and the average Q20 value reaches 97.76 % (Additional file 1: Table S2).

**Fig. 2:**
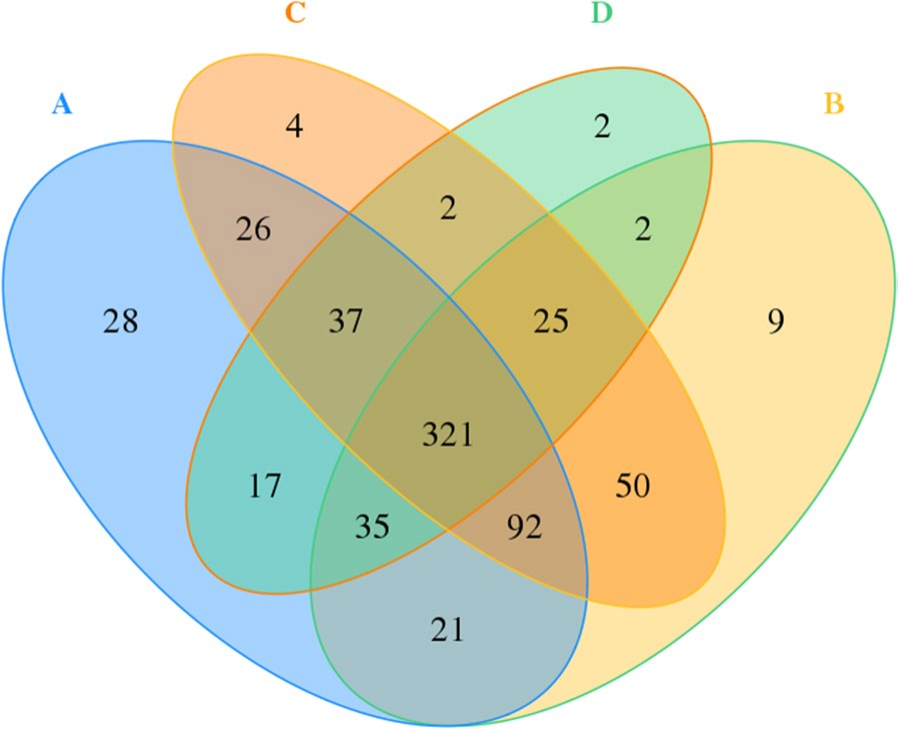
Venn diagram for calculated OTUs. Different groups are presented by different colors, the number of overlapping area among graphs with different colors is the number of OTU shared by multiple samples. Non-overlapping areas show count of unique OTU of each sample

The effective tags were clustered into OTU at a similarity level of 97%, and OTU representative sequences were annotated. The Venn diagram (Fig. 2) was used to show the number of shared and unique OTUs among the samples after clustering. A total of 671 OTUs were obtained, and 321 OTUs were shared by the four groups. Among them, the number of unique OTUs in group A was 28, group B was 9, group C was 4, and group D was 2.

### Estimation of gut microbial diversity

To verify whether the sequencing amount is sufficient for studying the microbial community, the randomly picked sequencing amount and the corresponding species number were used to construct rarefaction curve. The results revealed that all of them became relatively flat as the number of sequences analyzed increased, which indicated that the amount of sequencing was accurate and reasonable, and the depth of sequencing was enough to meet the experimental requirements (Additional file 1: Fig. S1a). To further confirm the reliability, the Shannon index curve was made according to the Shannon indexes of sequencing amount under different sequencing depths, which became flat with the number of sequences analyzed increased as well (Additional file 1: Fig. S1b). These results indicated that the majority of microorganisms in the samples had been captured, and the quality of the sequencing data was qualified for further analysis.

**Fig. 3:**
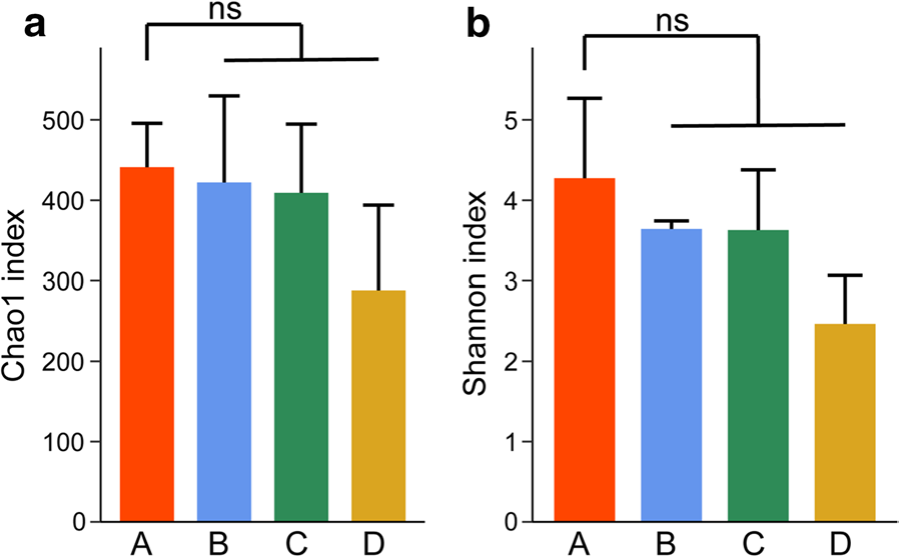
Alpha diversity metrics of fecal bacterial communities. **a** Histogram for comparison of species richness (Chao1 index) between the four groups. **b** Histogram for comparison of species diversity (Shannon index). (ns: not significant)

Subsequently, the Alpha diversity of microbial communities in the four groups of samples was measured based on the Chao1 estimators and the Shannon indexes, which represent species abundance and diversity, respectively. Compared with control group A, no significant differences were found in the Chao1 and Shannon indexes of the other three groups (Fig. 3). This result shows that our groups had no effect on the abundance and diversity of the microbial communities, however, they all declined gradually, which means that when purslane was increased to a certain amount, it may have an effect on the abundance and diversity of the gut microbiota.

### Microbial community composition and relative abundance

**Fig. 4:**
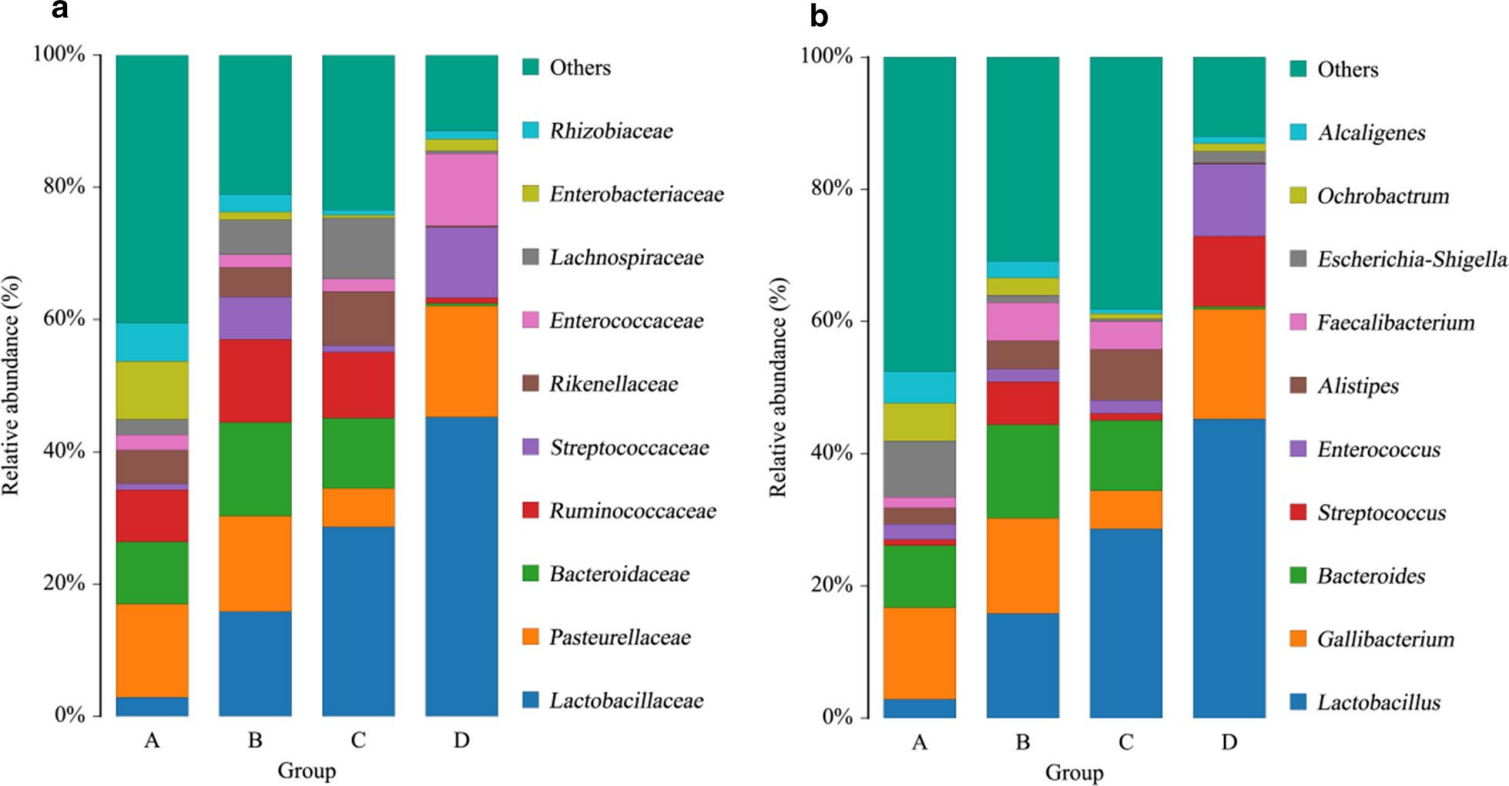
Histogram of the distribution of species. **a**, **b** Indicate the community composition of gut microbiota at the family and genus levels, respectively. The abscissa represents the group, and the ordinate represents the relative abundance. One color represents one species, the length of the color block present the relative richness proportion of the species. The figures show species with top ten richness levels, and the rest species are combined as “others” in the figures

The statistical results of OTUs indicated the relative abundance of bacteria at the taxonomic level of phylum, class, order, family, and genus. The relative abundance of species on family and genus level is shown in Fig. 4. The results showed that the bacterial composition and relative abundance of the four groups were different.

At the family level (Fig. 4a), the most predominant family in the control group A was *Pasteurellaceae*, followed by *Bacteroidaceae* and *Enterobacteriaceae*, while the other three groups were dominated by *Lactobacillaceae*, followed by *Pasteurellaceae* or *Bacteroidaceae*. Moreover, it is worth paying attention that the relative abundance of *Lactobacillaceae* in groups B, C and D containing purslane gradually increased with the increase of purslane, while *Enterobacteriaceae* rapidly decreased. Another interesting point was found that *Ruminococcaceae* and *Lachnospiraceae* had higher relative abundance in group B and C, but group D did not. In group D, the relative abundance of *Pasteurellaceae*, *Enterococcaceae* and *Streptococcaceae* were higher except for *Lactobacillaceae*, while the others were significantly lower.

The observation at the genus level was further implemented (Fig. 4b), and we found that the *Lactobacillus* displayed significant difference among the four groups of samples. Its relative abundance was only accounted for 2.85 % in group A, but it accounted for 15.83 %, 28.64 % and 45.28 % in groups B, C and D, respectively. In addition, in the control group A, the main microbial communities were *Gallibacterium* (13.87 %), *Bacteroides* (9.40 %) and *Escherichia-Shigella* (8.48 %). Remarkably, after purslane was fed, *Lactobacillus* became the most predominant genus, *Gallibacterium* did not change significantly, *Bacteroides* was significantly reduced in group D and replaced by *Streptococcus* and *Enterococcus*. Moreover, the *Escherichia-Shigella* enriched in group A only accounted for 1.08 %, 0.40 % and 1.68 % in groups B, C and D. For the remaining genera, it did not indicate significant differences in abundance compared with the control.

Taken together, the addition of purslane to feed broilers could interfere with the composition of the gut microbiota, which was mainly reflected in the effect on *Lactobacillus* of *Lactobacillaceae*. On the one hand, the increase in purslane led to an increase in the relative abundance of *Lactobacillus*. On the other hand, the abundance of *Escherichia-Shigella* in *Enterobacteriaceae* decreased rapidly with the increase in purslane.

### Overview of LEfSe analysis and function prediction of gut microbiota

**Fig. 5:**
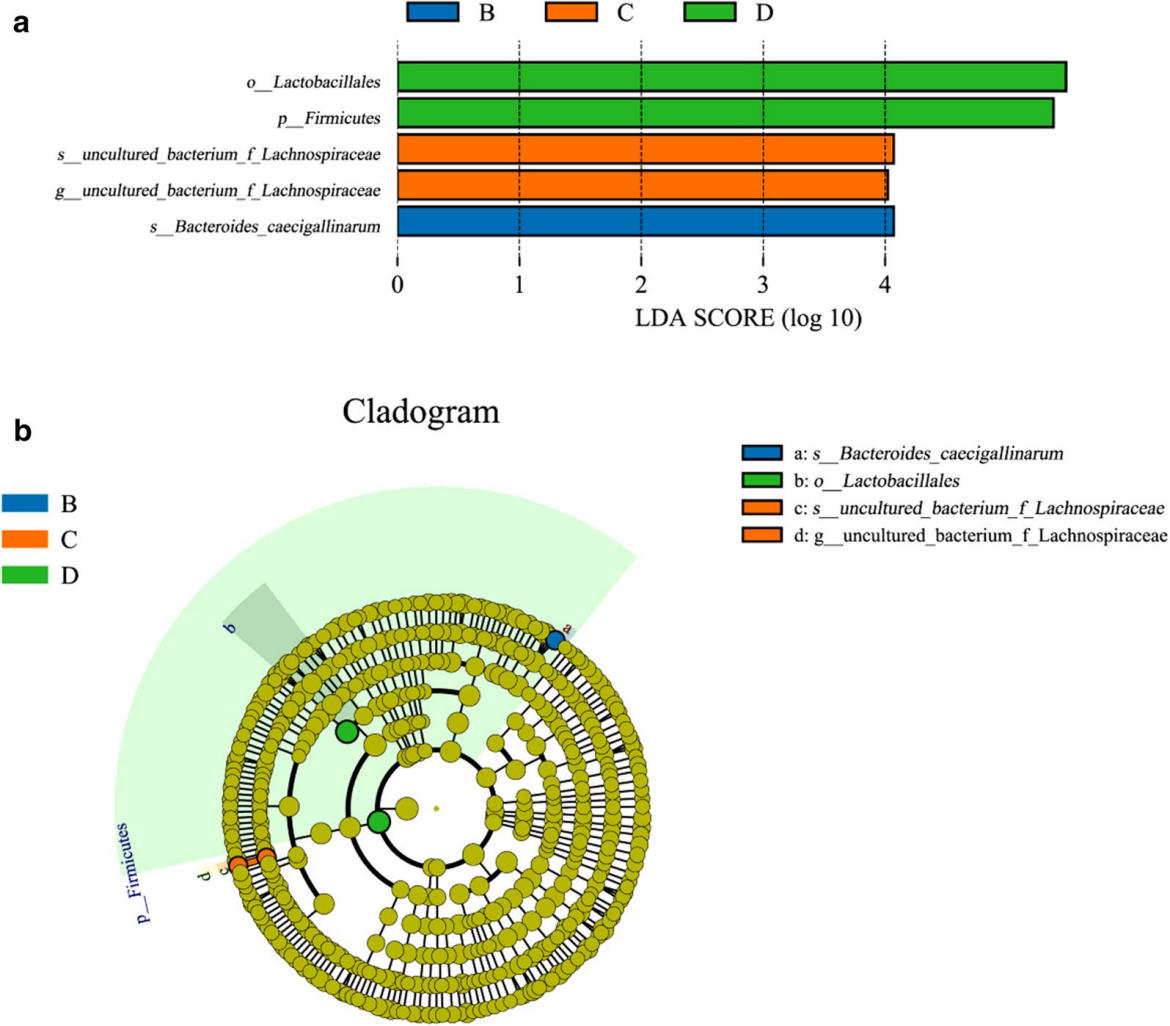
The differential taxa of gut microbiota after added purslane. **a** LDA value distribution histogram. **b** Cladogram of LEfSe analysis. The circle radiating from inside to outside of the cladogram represent the classification levels from phylum to species. Each small circle at different classification levels represents a classification at that level, and the diameter of the circle is proportional to the relative abundance. The blue, orange, and green circles represent the bacteria enriched in groups B, C and D, respectively, whereas the yellow circles represent the taxa with no significant difference

In order to find out which bacteria caused the statistical differences in the microbial communities between different groups, LEfSe analysis was used to identify biomarkers. According to the established Biomarker screening criteria (LDA score > 4), a total of five eligible species were identified (Fig. 5). As shown in Fig. 5a, the species with significant differences in group B was *Bacteroides_caecigallinarum*, group C was *uncultured_bacterium_f_Lachnospiraceae*, and group D was *Lactobacillales* and *Firmicutes*, which had great impact. The evolutionary branch graph of LEfSe analysis was depicted in Fig. 5b. The Cladogram shows that there were four differential bacterial taxa after added purslane. Specifically, group B had 1 species with significant difference, group C had 1 genus and 1 species with significant difference and both belonged to *Lachnospiraceae*, and group D had 1 phylum and 1 order with significant difference.

To infer the functional changes of the gut microbiota after added purslane, we used PICRUSt. Based on the KEGG database, a total of six biological metabolic pathways were revealed at level 1 pathway, included metabolism, environmental information processing, genetic information processing, cellular processes, human diseases and organismal systems. Meanwhile, the predicted secondary pathway was found to be mainly composed of 45 sub-functions, included Global and overview maps, carbohydrate metabolism, amino acid metabolism, membrane transport, metabolism of cofactors and vitamins, energy metabolism, nucleotide metabolism, translation, replication and repair and signal transport, etc. (Additional file 2: Table S3). Furthermore, the predicted KEGG Pathways differentially abundant between four groups, after purslane was fed, carbohydrate metabolism and nucleotide metabolism were enriched, and amino acid metabolism, metabolism of cofactors and vitamins, and energy metabolism were reduced. In addition, all sub-functions in the genetic information processing had a certain increase, while some had decreased in human diseases.

## Discussion

In the past few years, purslane has been widely used in various animal diets to improve animal growth and metabolism and disease resistance due to its high amounts of bioactive compounds. Abdel-Razek et al. (2019) showed that feeding purslane to Nile tilapia significantly increased the survival rate, and found plasmatic superoxide dismutase (SOD), catalase (CAT) and glutathione peroxidase (GPX) activities increased significantly with increase purslane levels as well. Zidan et al. (2014) showed that purslane effectively reduces the contents of serum total cholesterol (TC), triacylglycerols (TG) and low-density lipoproteins (LDL) accompanied with increases in high-density lipoproteins (HDL) in rats fed enriched-cholesterol diet, which contributes to anti-atherosclerosis. Beyond these, many studies on purslane in chickens have been widely reported. Aydin and Dogan (2010) indicated that adding purslane to the diet of laying hens significantly increased egg production and egg weight. Sadeghi et al. (2016) indicated that purslane significantly improved the feed efficiency and antioxidant status of broilers.

Gut microbiota plays a vital role for the growth and development of animals. Diet is a source of the composition of the gut microbiota. Dietary has profound effects on microbial composition, which in turn affects a series of metabolic, hormonal and neurological processes (Frame et al. 2020). Therefore, it is necessary to modulate the gut microbiota and promote its growth and development through the improvement of animal diets. Our study found that adding purslane to the diet has a significant effect on regulating the gut microbiota of broilers and enhancing their growth performance. Specifically, the body weight of broiler chickens increased with the increase of purslane. At 2 % and above, the weight gain was significantly higher, whereas the FCR was lower. In the investigation of the gut microbiota, it was found that the abundance and diversity of gut microbiota were slightly reduced, but there was no significant difference, which means that 3 % purslane may be the optimal content to enhance the growth performance of broilers under the premise of that the diversity of the gut microbiota is not reduced significantly.

Taking into account the difference in the amounts of purslane added, we compared the microbial communities of each group at each taxonomic level. The compositions of microbial communities are the main factors for the difference in growth performance. Our analysis showed that *Lactobacillus* of *Lactobacillaceae* was primary taxa after supplementation of diet with purslane, and accounted for a greater proportion than other differentially abundant taxa. This genus was one of the probiotics for humans and animals, which was of great significance to intestinal health. Some of the probiotics could increase the nutritional value of feed and were highly related to the animal growth performance, as well as the best alternatives to replacing antibiotics in the feed industry (Neveling and Dicks 2020). The probiotic *Lactobacillus* species might promote the maturation of the intestinal microbiota and enhancement of intestinal immunity against certain pathogens (Gao et al. 2017; Hardy et al. 2013). Additionally, *Lactobacillus* suggests a prominent role in improving chicken feed efficiency (Yan et al. 2017). *Lactobacillus* has been reported to lead to obesity or weight gain (Angelakis and Raoult 2010; Million et al. 2012). The results we obtained were in agreement with those reported, which also shows that it could promote nutrient absorption and energy extraction in the host.

Notably, the relative abundance of *Lactobacillus* was significantly enriched. In contrast, the abundance of *Escherichia-Shigella* in the *Enterobacteriaceae* was rapidly decreasing, which was also consistent with some studies (Sadeghi et al. 2016; Zhao et al. 2013). The vast majority of this genus are highly adapted pathogens, including species like Enterotoxigenic *Escherichia coli* that have been reported to cause diarrhea in piglets (Bin et al. 2018), and *Shigella* is a key pathogen causing bacillary dysentery (Phalipon and Sansonetti 2007). Interestingly, in the past, purslane was used to treat bacillary dysentery in China, and modern pharmacological studies have shown that purslane has a significant antibacterial effect on enteropathogenic pathogens, which may be due to the presence of a large number of flavonoids in purslane (Desta and Cherie 2018; Lei et al. 2015; Nayaka et al. 2014).

The species with the most significant differences in each group were identified through LEfSe analysis, and the results showed that *Lactobacillales*, *Firmicutes*, *Lachnospiraceae* and *Bacteroides_caecigallinarum* had a higher degree of influence. The *Lactobacillales* represents a morphologically, functionally and metabolically diverse group of bacteria, and lactic acid bacteria are the core of this group. This type of bacteria mediates most of the protective effects, such as anti-inflammatory by regulating the content of intestinal interleukin factor (Chih Tsai and Chou 2016; Mu et al. 2017). Obesity is associated with *Firmicutes*, a study suggested that the accumulation of fat was related to the increase in the abundance ratio of *Firmicutes*/*Bacteroidetes* (Turnbaugh et al. 2006). The significantly enriched taxa in this study belonged to the phylum of *Firmicutes*, which caused a significant increase, while the *Bacteroidetes* did not change much. The increase in the ratio of the two eventually led to weight gain in broilers. Meanwhile, the abundance of *Bacteroidetes* was found to be significantly reduced in the high-content purslane (3 %) group, which is why it has a more prominent effect on weight gain. Nevertheless, the bacteria within the *Lachnospiraceae* in *Firmicutes* had been reported to be potentially related to obesity as well as overweight (Kameyama and Itoh 2014; Tun et al. 2018). *Lachnospiraceae*, a family of obligate anaerobic bacteria, is involved in the production of short-chain fatty acids and the metabolism of bile acids to facilitate colonization resistance against intestinal pathogens, thereby maintaining the health of the host (Sorbara et al. 2020). The composition of the gut microbiota is closely linked to the intake of Omega-3 polyunsaturated fatty acids, particularly for bacteria of the *Lachnospiraceae* family (Costantini et al. 2017; Menni et al. 2017). At present, the role of *Bacteroides_caecigallinarum* has not been reported, and whether it affects obesity or weight gain remains poorly understood. Moreover, our data indicates that the weight gain of this group is not significant, which may be the result of low changes in the intestinal microbiota due to the low content of purslane.

The ability to convert food into body weight is of interest to the animal production industries, and the gut microbiota play an important role in the efficiency of energy extraction from diets and host metabolism (Stanley et al. 2012, 2013). PICRUSt analysis revealed changes in metabolism caused by these bacteria, suggesting that the carbohydrate metabolism pathway is the primary factor that promotes growth. Accumulating evidence indicates that *Lactobacillus* could improve host physiology and metabolism and has significant effects on weight modification (Drissi et al. 2017; Mountzouris et al. 2007). The bacteria of the genus *Lactobacillus* possess numerous carbohydrate transport systems to regulate carbon metabolism (Monedero et al. 2007). Carbohydrates are the main component and main energy supply substance of the structure of life cells. Abundant carbohydrate metabolism indicates stronger carbon source transportation or metabolism. The existence of *Lactobacillus* contributes to the expression of glycogen metabolism genes and glycogen accumulation, thereby increasing the capacity of harvesting energy from the diet, thus promoting more efficient absorption of calories and subsequent weight gain (Goh and Klaenhammer 2014).

In this study, the increase in relative abundance of *Lactobacillus* and the decrease in *Escherichia-Shigella* may be the most critical reasons for weight gain. Certain bacteria of these genera such as *Lactobacillus paracasei* and *Escherichia coli* could increase intracellular fat storage and enhance lipid catabolism, respectively (Tazi et al. 2018). It means that the increase *Lactobacillus* contributes to store fat, and the decrease of *Escherichia* inhibits lipids secretion, which is ultimately reflected in the body weight gain. For other taxa, such as *Ruminococcaceae* and *Lachnospiraceae*, they have also been found to contain key carbohydrate active enzymes, sugar transport mechanisms and metabolic pathways, so that they are more specialized for the degradation of complex plant material (Biddle et al. 2013), which may be one of the reasons for the significant enrichment in animal intestines after feeding purslane. In our study, the weight gain of the C (2 %) and D (3 %) groups was significant, and they were consistent in terms of the increase in the relative abundance of beneficial bacteria and the decrease in pathogenic bacteria. These findings, in accordance with the previous reports that gut microbiota is improved by purslane, might contribute to concluding that the effects of purslane on weight gain might depend on its modulation effect on gut microbiota.

In conclusions, this study demonstrated that the intake of 2 % and 3 % purslane could significantly increase the body weight gain and lead to a reduction in FCR. Moreover, purslane-fed increased the relative abundance of beneficial bacteria in the chicken intestines. What’s more, purslane-fed drastically modified the gut microbial species at the family level, increasing the gut microbiota associated with obesity or weight gain, such as *Lactobacillaceae* and its genus *Lactobacillus*. Meanwhile, purslane-fed decreased the relative abundance of harmful bacteria that has been reported to be related to diseases, such as *Escherichia-Shigella* in family *Enterobacteriaceae*. Our findings indicated that the potential applications of purslane in the body weight gain might closely depend on its modulatory effect on gut microbiota. In short, adding appropriate purslane to broiler diets could increase the abundance of *Lactobacillus* in the gut microbiota and promote growth performance, thereby producing safer and healthier livestock products.

## Supplementary information

**Additional file 1: Table S1.** Composition and nutrient levels of basal diets for broilers. **Table S2.** Statistics of sample sequencing data processing result. **Figure S1.** Rarefaction curve and Shannon index curve drawn based on sequencing data.

**Additional file 2: Table S3.** Predicted KEGG Pathways differentially abundant between four groups.

## Data Availability

All raw sequences were submitted to the NCBI Sequence Read Archive (https://www.ncbi.nlm.nih.gov/sra/) under BioProject PRJNA670355. The data supporting the conclusion of this article are included in this article.
