# Supplementary material for: Dietary purslane (Portulaca oleracea L.) promotes the growth performance of broilers by modulation of gut microbiota
Source: AMB Express. 2021 Feb 23;11:31. doi: 10.1186/s13568-021-01190-z (PMC7902751; doi:10.1186/s13568-021-01190-z)

**Table S1.** Composition and nutrient levels of basal diets for broilers

| **Composition** | **Content** | **Nutrient levels** | **Content** |
| --- | --- | --- | --- |
| Corn | 67% | Metabolizable energy (MJ/kg) | 12.56 |
| Soybean meal | 29% | Crude protein (%) | 18.57 |
| Premix | 4% |  |  |

Note: The premix provided the following per kg of diets: VA 125000-250000IU, VD_3_ 37500-125000IU, VE 500mg, VK_3_ 33mg, VB_1_ 37mg, VB_2_ 125mg, VB_6_ 56mg, choline chloride 7.5g, biotin 2.5mg, pantothenic acid 225mg, nicotinic acid 750mg, folic acid 21mg, Zn 1.2-3g, Mn 1.2-3.7g, Se 3.7-12.5mg, Cu 0.12-0.625g, Fe 1.2-3.0g, Ca 10-30% and total P 3.0%.

**Table S2.** Statistics of sample sequencing data processing result

| Sample ID^a^ | Raw Tags | Clean Tags | Effective Tags | AvgLen (bp)^b^ | GC(%)^c^ | Q20(%)^d^ |
| --- | --- | --- | --- | --- | --- | --- |
| A1 | 77696 | 74562 | 68307 | 420 | 52.97 | 97.69 |
| A2 | 77801 | 74477 | 70749 | 424 | 53.69 | 97.63 |
| A3 | 77659 | 74419 | 67512 | 419 | 53.06 | 97.61 |
| B1 | 78232 | 75752 | 66930 | 418 | 52.27 | 97.9 |
| B2 | 77688 | 74706 | 71890 | 422 | 51.93 | 97.69 |
| B3 | 78079 | 75043 | 71980 | 423 | 53.22 | 97.75 |
| C1 | 77826 | 74768 | 70043 | 418 | 52.31 | 97.67 |
| C2 | 78633 | 76096 | 66370 | 425 | 51.44 | 97.91 |
| C3 | 78148 | 75396 | 71734 | 421 | 52.75 | 97.8 |
| D1 | 78603 | 75847 | 68530 | 429 | 52.32 | 97.77 |
| D2 | 78446 | 75580 | 69298 | 427 | 51.53 | 97.74 |
| D3 | 78664 | 76135 | 73685 | 424 | 52.22 | 97.94 |
| Total | 937475 | 902781 | 837028 |  |  |  |
| Mean | 78123 | 75232 | 69752 | 423 | 52.48 | 97.76 |

^a^ Sample ID: A1~3 are 3 samples of group A, B1~3 are 3 samples of group B, C1~3 are 3 samples of group C, and D1~3 are 3 samples of group D.

^b^ AvgLen (bp) is the average sequence length of sample.

^c^ GC(%) is the GC content of sample, i.e. the percentage of G and C bases in all bases.

^d^ Q20(%) is the percentage of bases which quality value is no less than 20 in all bases.

**Fig. S1** Rarefaction curve and Shannon index curve drawn based on sequencing data


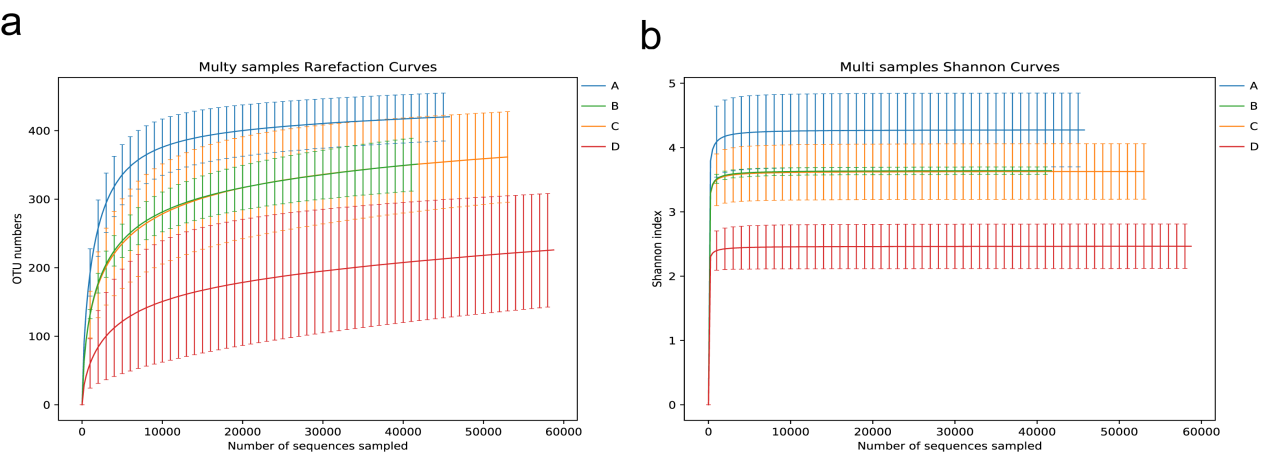

Supplement: Supplementary file 1 — Additional file 1: Table S1. Composition and nutrient levels of basal diets for broilers. Table S2. Statistics of sample sequencing data processing result. Figure S1. Rarefaction curve and Shannon index curve drawn based on sequencing data. [file 13568_2021_1190_MOESM1_ESM.docx]
